# Supplementary material for: Genetic diversity analysis of the natural regeneration loci of Liriodendron chinense in artificial mixed forests in the rocky desertification area of Western Hunan
Source: PeerJ. 2025 Oct 23;13:e20138. doi: 10.7717/peerj.20138 (PMC12554308; doi:10.7717/peerj.20138)
Supplement: Supplemental Information 2 [file peerj-13-20138-s002.docx]

**Supplement information for Materials & Methods**

**TP-M13-SSR polymerase chain reaction (PCR) method**

The TP-M13 forward primer is the 5 'end of the forward primer with M13 tail (5 TGTAAAACGACGGCCAGT-3), and the 5 'end of the M13 fluorescent forward primer was labeled with ROX, HEX, FAM and TAMRA. The total volume of the reaction system was 10*μl*, which included 2.35*μl* of ddH2O, 5*μl* of 2×Rapid Taq Master Mix, 0.25*μl* of F-Primer, 0.25*μl* of R-Primer, 0.25*μl* of fluorescent primer, and 1.9*μl* of Template DNA. Touch-down PCR was used for amplification, with the PCR amplification consisting of 1 cycle at 94°C for 4 minutes (pre-denaturation step), followed by 94°C for 15 seconds, 60°C for 15 seconds, with a decrease of 0.7°C per cycle, and 72°C for 30 seconds for a total of 15 cycles (annealing step). This was followed by 94°C for 15 seconds, 49.5°C for 15 seconds, and 72°C for 30 seconds for a total of 15 cycles (general amplification), with a final extension at 72°C for 20 minutes, and then stored at 4°C.

**Specific information for parentage analysis**

This experiment employs this method for parentage analysis of the four diameter classes of the *L. chinense*, with the specific classification method as follows: the previous diameter class serves as the offspring, and the sum of the remaining diameter class plants serves as the parents for analysis. Specifically, the candidates for Class I are the *L. chinense* from Classes II, III, IV, and V; the candidates for Class II are from Classes III, IV, and V; the candidates for Class III are from Classes IV and V; and the candidates for Class IV are from Class V. The parentage analysis simulation parameters are set as follows: the number of offspring for each class is set as 126, 124, 28, and 38, the number of candidate par-ents is set as 192, 68, 40, and 2, the simulation calculation is set for 10,000 cycles, with a candidate parent ratio of 1, a locus pairing rate of 0.99, an average genotype error rate of 0.01, and a reference confidence level of 80% to 95%.
